# Interventions to improve adolescents’ sense of coherence and social support on quality of life and gingivitis: a cluster-randomised clinical trial

**DOI:** 10.1007/s11136-026-04232-w

**Published:** 2026-06-05

**Authors:** Mario Vianna Vettore, Gabriela de Figueiredo Meira, Ana Virgínia S. S. Castilho, Rharessa Gabrielly Mendes, Fernanda Ruffo Ortiz, Camila Silveira Sfreddo, Thiago Machado Ardenghi, Silvia Helena de Carvalho Sales Peres

**Affiliations:** 1 Department of Dentistry and Oral Health, Aarhus University, Aarhus, Denmark; 2 Department of Pediatric Dentistry, Orthodontics and Public Health, Bauru School of Dentistry - University of São Paulo, Alameda Octávio Pinheiro Brisolla, 9-75, Bauru, Brazil; 3 Atitus Educação, Passo Fundo, Brazil; 4 Department of Semiology and Clinic, Faculty of Dentistry, Federal University of Pelotas, Pelotas, RS Brazil; 5 Department of Stomatology, School of Dentistry, Federal University of Santa Maria, Santa Maria, RS Brazil

**Keywords:** Oral health promotion, Salutogenesis, Sense of coherence, Salutogenesis

## Abstract

**Purpose:**

to evaluate two interventions to improve sense of coherence (SOC) and social support on oral health-related quality of life (ORHQoL), health-related quality of life (HRQoL), dental biofilm and bleeding on probing (BOP) among adolescents.

**Methods:**

A cluster-randomised school-based clinical trial was conducted with 254 adolescents aged 15–16 years from four secondary schools in Brazil. The SOC intervention group (SOC-G) included seven 60-minute sessions aimed at developing and empowering positive attitudes, personal skills and coping strategies. The SOC plus social support intervention group (SOC/SS-G) comprised face-to-face and online meetings between participants and high school students to enhance social support, along with the SOC intervention.

**Results:**

Mixed-effects models revealed significant effects of SOC-G and SOC/SS-G on SOC, social support, self-esteem, dental biofilm, BOP, OHRQoL and HRQoL at three-month follow-up. Structural equation modelling showed direct effects of interventions on dental biofilm and HRQoL. Interventions indirectly predicted ORHQoL via SOC change. Social support change mediated the effect of the interventions on BOP.

**Conclusion:**

The present findings suggest that interventions aiming to enhance SOC and social support can positively impact quality of life and periodontal clinical outcomes among adolescents.

**Brazilian register of clinical trials (ReBEC):**

Identifier: RBR-3rxj6y6.

**Supplementary Information:**

The online version contains supplementary material available at 10.1007/s11136-026-04232-w.

## Abstract (plain language summary)

Adolescents living in deprived areas often face challenges that affect their oral health and quality of life. Improving their ability to cope with stress and strengthening social support may help improve both their quality of life and oral health. This study evaluated whether interventions that build coping skills and social support can improve adolescents’ oral health and quality of life. The study tested two school-based programs: One focused on coping skills, and another combining coping skills with individual peer support to see if they improve quality of life and oral health in teenagers. Both programmes led to better coping, more social support, higher self-esteem, and improvements in oral health (less dental plaque and gum bleeding). These changes also improved how teens felt about their health and well-being. The results suggest that helping teens build resilience and social connections can lead to real health benefits to their oral health and quality of life.

**Supplementary Information:**

The online version contains supplementary material available at 10.1007/s11136-026-04232-w.

## Introduction

Health promotion approaches encompass a range of multidisciplinary strategies that use complementary actions to enable individuals to exert more control and responsibility over personal and environmental factors that influence their health [1]. Effective oral health promotion should create supportive environments that encourage people to adopt healthy choices, considering the mediating factors between individuals and their various environments [2].

The impact of health promotion interventions on oral health has been evaluated using subjective and clinical outcome measures [3, 4]. Combining self-reported and normative outcomes aligns with the contemporary concept that oral health is multifaceted, encompassing physical function (e.g., ability to speak and smile), symptoms (e.g., absence of pain), and emotional (e.g., emotional well-being) and social aspects (e.g., social participation) [5]. A relevant patient-reported outcome used in previous interventions is oral health-related quality of life (OHRQoL) [4, 6, 7]. OHRQoL is a multidimensional construct reflecting how oral health impacts a person’s overall well-being and ability to perform daily activities [8, 9].

Gingivitis is considered a common oral condition during adolescence that is associated with worse self-rated oral health and poor OHRQoL [10–12]. The oral impacts of gingivitis are possibly related to clinical symptoms, such as edema and marginal bleeding, that can affect oral aesthetics and function [10–12]. Gingival status and OHRQoL can be affected by psychosocial factors [11, 13, 14]. Previous studies have shown that self-esteem, oral health beliefs, religiosity and happiness predicted better gingival status in adolescents [13, 15]. In addition, self-esteem, oral health beliefs and sense of coherence (SOC) were associated with better OHRQoL [11, 14]. Therefore, interventions addressing psychosocial factors have the potential to enhance the effectiveness of oral health promotion interventions.

Intervention studies in oral health promotion aimed at reducing dental biofilm levels and improving gingival status among adolescents revealed promising but limited results [3, 16]. This might be explained because a large number of community-based and individualized oral health promotion interventions were predominantly top-down and knowledge-based using health behaviour change theory as framework [3, 16]. Oral health education has been combined with diverse interventions in previous intervention studies, including clinical preventive measures (e.g., fissure sealants, dental prophylaxis) [17, 18]. However, controlled trials focused on enhancing psychosocial factors to improve oral health status are scarce [6, 7]. Two school-based cluster-randomised trials to increase SOC and OHRQoL were conducted in children and young adolescents and evaluated the impact of the intervention after three months [6, 7]. A comprehensive intervention involving schoolchildren 10- to 12-year-olds aimed to support the participants to think positively about their health, increase their knowledge and awareness of health, self-esteem, self-efficacy, and develop positive attitudes, personal skills, and coping strategies [6]. A similar intervention was tested in socially vulnerable children and adolescents who received a school-based oral health promotion intervention [7]. These interventions positively influenced changes in SOC, OHRQoL and gingival status [6, 7].

As far as the authors are aware, no study evaluated the effect of interventions to improve SOC and social support, and the possible impact of such interventions on OHRQoL, health-related quality of life (HRQoL), dental biofilm, and gingival status in adolescents. Adolescence is a meaningful transition period in a person’s life, marked by changes in biology, behaviours, and psychological status that are influenced by social environmental conditions [19]. Therefore, this school-based clinical trial aimed to test the effectiveness of two school-based interventions to improve SOC and social support on ORHQoL, HRQoL and gingival health among underprivileged adolescents. Furthermore, we explored the pathways by which the interventions may improve OHRQoL, HRQoL, and gingival health, assessing the direct and indirect pathways among psychosocial and clinical variables. It was hypothesised that SOC intervention will lead to better adolescent’s subjective and periodontal clinical outcomes. It was also conjectured that the intervention targeting both SOC and social support would yield additional improvement in quality of life and periodontal outcomes.

## Methods

The study is presented in accordance with the Consolidated Standards of Reporting Trials (CONSORT) checklist [20]. The trial has been registered in the Brazilian Register of Clinical Trials (ReBEC), Protocol Registration and Results System under identifier RBR-3rxj6y6.

### Trial design and participants

A double-stage, cluster-randomised clinical trial was conducted in the city of Bauru, São Paulo, Brazil. Bauru is a mid-sized, developed city with a Human Development Index of 0.801, located in the interior of São Paulo state. As of 2024, Bauru is the most populous city in the center-west region of the state, with approximately 388,011 inhabitants [21].

The study included students aged 15 and 16 years selected from classrooms in four public secondary schools randomly selected, and located in two socially deprived areas (North and West regions) as they had the greater number of families living in extreme poverty in the city (per capita family income up to EUR 13.8) [22]. Adolescents using orthodontic appliances and pregnant or breastfeeding students were excluded, as these conditions may affect oral health and self-perception, which could compromise data collection.

## Interventions

Participants were randomly allocated to two intervention groups and a control group (CG). The SOC intervention group (SOC-G) aimed to enhance adolescent’s sense of coherence (SOC), and the SOC plus social support intervention group (SOC/SS-G) focused on strengthening adolescents’ SOC and social support.

The intervention designed to enhance adolescents’ SOC was group-based and consisted of 60-minute sessions delivered once a week carried out over 7 consecutive weeks based on previous studies [6, 7]. Initially, three dentists and one psychologist previously trained received a manual outlining the objectives and instructions of the intervention. All activities were conducted in a private room in each school. The SOC-G activities were based on the salutogenesis theory that aimed to encourage adolescents to think positively about their health, increase their knowledge and raise awareness of factors influencing mental health, oral health, and self-esteem [23, 24]. These activities also focused on developing positive attitudes, personal skills, and coping strategies, which are components of SOC and principles of participation and empowerment. In addition, the activities also sought to increase students’ perception of risk behaviours, such as the use of illicit substances.

The SOC/SS-G included the activities already described to enhance SOC and an additional component to increase perceived social support. The social support intervention was based on the social cognitive views of social support that are concerned primarily with the perception of support [25]. A major tenet is that stable beliefs about support are shaped to conform to preexisting beliefs after a person forms consistent opinions about how supportive other people are. The social-cognitive perspective emphasises generalised beliefs about the supportiveness of others and predicts that perceived support promotes self-esteem, which leads to health outcomes [25]. The social support intervention was individually based and carried out by student-tutors who worked with the participating adolescents. The student-tutors were high school adolescents with high academic performance and who stood out in complementary after school activities. The intervention consisted of an initial face-to-face meeting between tutors and students held in person at schools so that they could get to know each other and establish an initial emotional bond. Subsequent six meetings were held virtually via WhatsApp, where students were encouraged to discuss their feelings and any issues related to school, teachers, relatives, and friends. Students could also ask for support from tutors to help with school activities.

Adherence to interventions was recorded using attendance records. The feasibility of implementing SOC-G in real-world school settings appears to be high, as it is a group-based intervention conducted through structured sessions and utilises existing school facilities. In contrast, the feasibility of implementing SOC/SS-G intervention can be considered moderate it requires additional resources, including the recruitment of student tutors and digital engagement tools.

## Data collection

Participants completed the questionnaires individually using Google Forms and underwent dental examinations at baseline (March to November 2024) and three months after intervention, in a private room at each school. Dental examinations were conducted by two calibrated examiners using biosafety equipment. A plain dental mirror and a WHO periodontal probe were used to perform the examinations under natural light. Participants were examined while seated in a well-lit and ventilated location.

## Outcomes

The outcomes were evaluated at baseline and three months after the interventions. A three-month post-intervention follow-up was selected to reduce social desirability bias associated with immediate assessments and due to feasibility and practicality reasons, minimising excessive burden on participants and schools. OHRQoL and HRQoL were the primary outcomes. OHRQoL was measured using the Child Perception Questionnaire 11–14 (CPQ 11–14) [26, 27], consisting of 16 items grouped into four domains: oral symptoms, functional limitation, social well-being, and emotional well-being. Five response options are used for each question: 0 = ‘never’, 1 = ‘once or twice’, 2 = ‘sometimes’, 3 = ‘frequently’ and 4 = ‘every day/almost every day’. Higher CPQ 11–14 scores indicate worse OHRQoL. HRQoL was assessed using the Kiddo-KINDL questionnaire [28, 29]. Kiddo-KINDL is a 24-item questionnaire based on a 5-point Likert scale with the following response options: 1 = ‘never’, 2= ‘rarely’, 3 = ‘sometimes’, 4 = ‘often’ and 5 = ‘always’. Six subscales corresponding to HRQoL dimensions include physical well-being, emotional well-being, self-esteem, family, friends and daily routine/school. The scores of negative items are reversed before calculating the total overall and dimension scores.

Dental clinical measures of gingivitis and dental biofilm were the secondary outcomes assessed according to the number of sites with bleeding on probing (BOP) and dental biofilm (Visible Plaque Index) assessed at six sites per tooth [30, 31]. In addition, BOP measures were used to classify participants as with healthy gingival status (BOP score < 10%) or localized/generalized gingivitis (BOP score ≥ 10%) for descriptive purposes [32].

## Variables

Demographic variables included sex (male or female). Maternal schooling assessed according to the total number of years of schooling with approval was categorised as ‘1 to 8 years’ (primary education), ‘9 to 12 years’ (secondary education), and ‘13 or more’ (higher education).

Dental caries was measured according to the number of permanent teeth with clinical cavities caused by caries, based on the decayed component of the Decayed, Missing, and Filled Teeth (DMFT) index [33].

Sense of coherence scale (SOC-13) was used to measure adolescents’ SOC [23, 34]. The 13 items were answered on a 5-point Likert scale. The total SOC score was determined by adding up the scores of all 13 items, and may vary from 16 to 65. Higher SOC scores indicate stronger SOC. Social support was assessed using the Social Support Appraisals (SSA) questionnaire [35, 36]. SSA consists of 30 items grouped in four dimensions: family, friends, teachers and others. Responses follow a 6-point Likert scale. The total score ranges from 30 to 180 points, and the higher the score, the greater the perceived social support.

Self-esteem was measured using the Rosenberg Self-Esteem Scale (RSES) [37, 38]. The scale comprises 10 items responded using a 4-point Likert scale. The scores of negative items are reversed before obtaining the total score that can range from 0 to 30. The higher the score, the greater the self-esteem.

Cross-culturally adapted versions of the scales for Brazilian population were used.

### Reliability study

Before baseline data collection, 20 adolescents were interviewed to assess their understanding and the internal consistency of the questionnaires. No changes in the questionnaires’ items were needed. Cronbach’s alpha coefficients ranged from 0.808 (HRQoL three months after intervention) to 0.906 (SOC baseline) (Table S1).

## Sample size calculation

The sample size calculation was based on detecting differences in mean CPQ 11–14 scores among three groups (two intervention arms and one control) with equal allocation (1:1:1 ratio). Using estimates from Nammontri et al. [6] (control mean = 24.32, intervention mean ≈ 18.53, SD ≈ 15.5), the effect size (Cohen’s f) was calculated as 0.19. For a one-way ANOVA with three groups, 95% confidence level, and 80% power, the required sample size was approximately 73 participants per group. To account for potential attrition, we aimed to recruit at least 80 participants per group, resulting in a total sample size of 219 participants. The sample size was calculated using the statistical calculator available on https://www.statskingdom.com/sample_size_regression.html.

## Randomisation

The randomisation process was conducted in two stages to maintain the integrity of the cluster-randomised controlled trial. First, the total number of eligible public schools in the North and West regions of Bauru was determined (*n* = 22). Four schools were randomly selected using the WinPepi Software by a researcher not involved in the data collection (SP). These schools were similar in size (approximately 180 to 300) and academic performance (Brazilian School Performance Index) [39]. After selection, the four schools were randomly allocated to three study arms: one control group and two intervention groups. Because two of the schools were large and two were small, the smaller schools were grouped together for allocation purposes to maintain balance. Randomisation was performed using WinPepi, ensuring allocation concealment. Within each school, eligible classrooms (Year 10 and Year 11, students aged 15–16 years) were identified. From these, classrooms were randomly selected to participate: four classrooms from the two control schools, two classrooms from the first intervention school, and two classrooms from the second intervention school. All students in the selected classrooms were invited to participate in the study.

**Table 1.** Baseline characteristics of the control and intervention groups.

Variables	Control group (*n* = 86)	SOC-G (*n* = 81)	SOC/SS-G (*n* = 87)	*P* value
Sex, n (%)				0.41
Male	48 (55.8)	38 (46.90)	41 (47.10)	
Female	38 (44.2)	43 (53.10)	46 (52.90)	
Mother’s education, n (%)				0.89
1–7 years	45 (52.3)	42 (51.90)	49 (56.30)	
8–11 years	28 (32.6)	29 (35,80)	25 (28.70	
≥ 12 years	13 (15.1)	10 (12.30)	13 (14.90)	
SOC, mean (SD)	38.3 (7.54)	36.77 (8.19)	37.15 (8.21)	0.38
Social Support, mean (SD)	112.56 (19.21)	119.54 (16.92)	113.98 (13.37)	0.93
Self esteem, mean (SD)	17.03 (5.02)	17.28 (6.00)	17.25 (5.22)	0.83
Dental caries, mean (SD)	2.09 (2.63)	2.04 (2.54)	2.22 (2.55)	0.74
Dental biofilm, mean (SD)	24.57 (26.78)	27.33 (22.94)	20.78 (18.85)	0.65
Gingival status, mean (SD)	13.86 (21.71)	16.99 (24.25)	10.44 (16.42)	0.14
Gingivitis, n (%)				0.33
No	55 (64.90)	48 (59.30)	61 (70.10)	
Yes	31 (36.00)	33 (40.70)	26 (29.90)	
OHRQoL, mean (SD)	16.94 (9.83)	16.63 (9.45)	16.48 (10.78)	0.93
HRQoL, mean (SD)	53.41 (11.88)	60.17 (13.28)	59.08 (13.37)	0.002

SOC-G: intervention group aimed to enhance adolescent’s sense of coherence. SOC/SS-G: intervention group aimed to enhance adolescent’s sense of coherence and social support.*P* value refers to chi-square test (categorical variables) and Kruskal-Wallis (continuous variables).

### Blinding

Participants were informed about their allocation group only after baseline data collection. Examiners who collected pre- and post-intervention clinical data were blinded to participant’s group allocation. The researcher who conducted the data analysis did not participate in the data collection and interventions and was blinded to group allocation.

### Statistical analysis

Data analysis was performed in four steps using SPSS software (Statistical Package for Social Sciences) version 22.0 and AMOS SPSS version 29.0. Baseline data between control and intervention groups were compared using Chi-square test for categorical variables and Kruskal-Wallis test continuous variables. Pairwise comparisons of SOC, social support and self-esteem within groups between baseline and 3-month post intervention were assessed using Wilcoxon signed rank test.

Mixed-effects models were used to evaluate the effect of the interventions through comparing SOC, social support, self-esteem, OHRQoL, HRQoL, dental biofilm and gingival status scores between baseline and three months after intervention adjusted for baseline outcome measures. The fixed effect was group allocation, and mixed effect was schools, respectively. The Bonferroni test adjusted for multiple comparisons was used for post hoc pairwise analyses.

The minimally important difference (MID) was calculated for SOC, social support, self-esteem, dental biofilm, gingivitis, OHRQoL and HRQoL using the distribution-based approach to estimate effect size for the smallest change in a score considered meaningful from the patient’s or clinician’s perspective [39]. The effect size is expressed in standard deviation units, with the following benchmarks used for interpretation: small (0.2), moderate (0.5) and large (0.8) effect [40].

**Table 2.** Changes in SOC, social support, oral clinical measures and quality of life between the three groups at baseline and three months after intervention

Variables	Baseline, mean (SD)	3 months after intervention, mean (SD)				
	Control group	SOC-G	SOC/SS-G	Control group	SOC-G	SOC/SS-G
SOC	38.3 (7.54)	36.77 (8.19)	37.15 (8.21)	35.48 (8.21)**	41.20 (8.86)**	42.20 (7.05)
Social Support	112.56 (19.21)	119.54 (16.92)	113.98 (13.37)	111.40 (22.25)**	128.70 (20.23)**	126.52 (15.17)
Self-esteem	17.03 (5.02)	17.28 (6.00)	17.25 (5.22)	13.23 (3.23)**	20.54 (4.89)**	20.56 (4.46)
Dental biofilm	24.57 (26.78)	27.33 (22.94)	20.78 (18.85)	26.37 (28.13)**	18.42 (16.41)**	15.77 (16.56)
Gingivitis	13.86 (21.71)	16.99 (24.25)	10.44 (16.42)	15.26 (23.30)**	9.94 (16.81)**	7.67 (13.76)
OHRQoL	16.94 (9.83)	16.63 (9.45)	16.48 (10.78)	18.03 (8.41)**	13.80 (8.60)**	13.14 (7.77)
HRQoL	53.41 (11.88)	60.17 (13.28)	59.08 (13.37)	34.30 (9.49)**	65.99 (12.96)**	72.53 (8.10)

SOC-G: intervention group aimed to enhance adolescent’s sense of coherence. SOC/SS-G: intervention group aimed to enhance adolescent’s sense of coherence and social support. P values refer to post hoc Bonferroni test adjusted for multiple comparisons **p < 0.01.

**Fig. 1:**
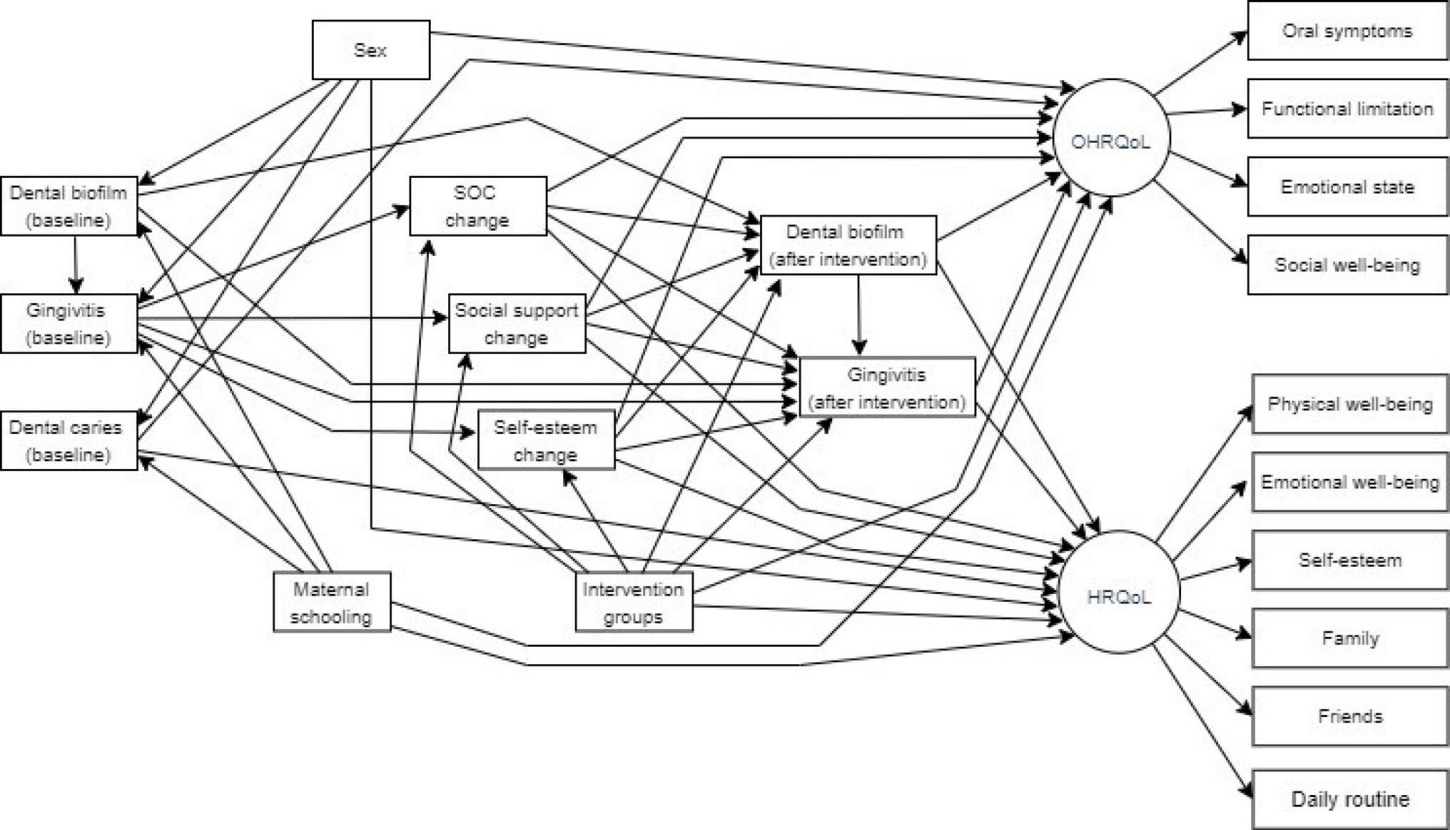
Full theoretical model linking intervention groups, clinical measures, psychosocial factors and quality of life measures

Confirmatory factor analysis (CFA) was performed to test the hypothesized measurement model comprising the latent variables OHRQoL and HRQoL. Structural equation modelling (SEM) simultaneously tested the predictors of OHRQoL, HRQoL, dental biofilm and gingival status at follow-up according to a theoretical model (Fig. 1). Direct and indirect effects and 95% confidence intervals (CIs) were estimated by bias-corrected bootstrapping with 900 replications using maximum likelihood estimation. Statistical significance of indirect effects and 95% CIs were used to assess mediation between variables. After testing the full model, nonsignificant paths and variables were removed, and the model was re-estimated to obtain a statistically parsimonious model. The full and parsimonious models were compared using the chi-square test. The following fit indices were used to assess model fit: χ_2_ /degrees of freedom (df) ratio < 3, root mean square error of approximation (RMSEA) < 0.06, standardized root mean square residual (SRMR) < 0.08, goodness-of-fit index (GFI) > 0.90, and comparative fit index (CFI) > 0.90.

### Ethical aspects

This study followed the protocol established by the Declaration of Helsinki (published in 1975 and revised in 2013) and was approved by the Research Ethics Committee of the Bauru School of Dentistry, University of São Paulo, protocol number: 74258423.0.0000.5417.

## Results

**Fig. 2:**
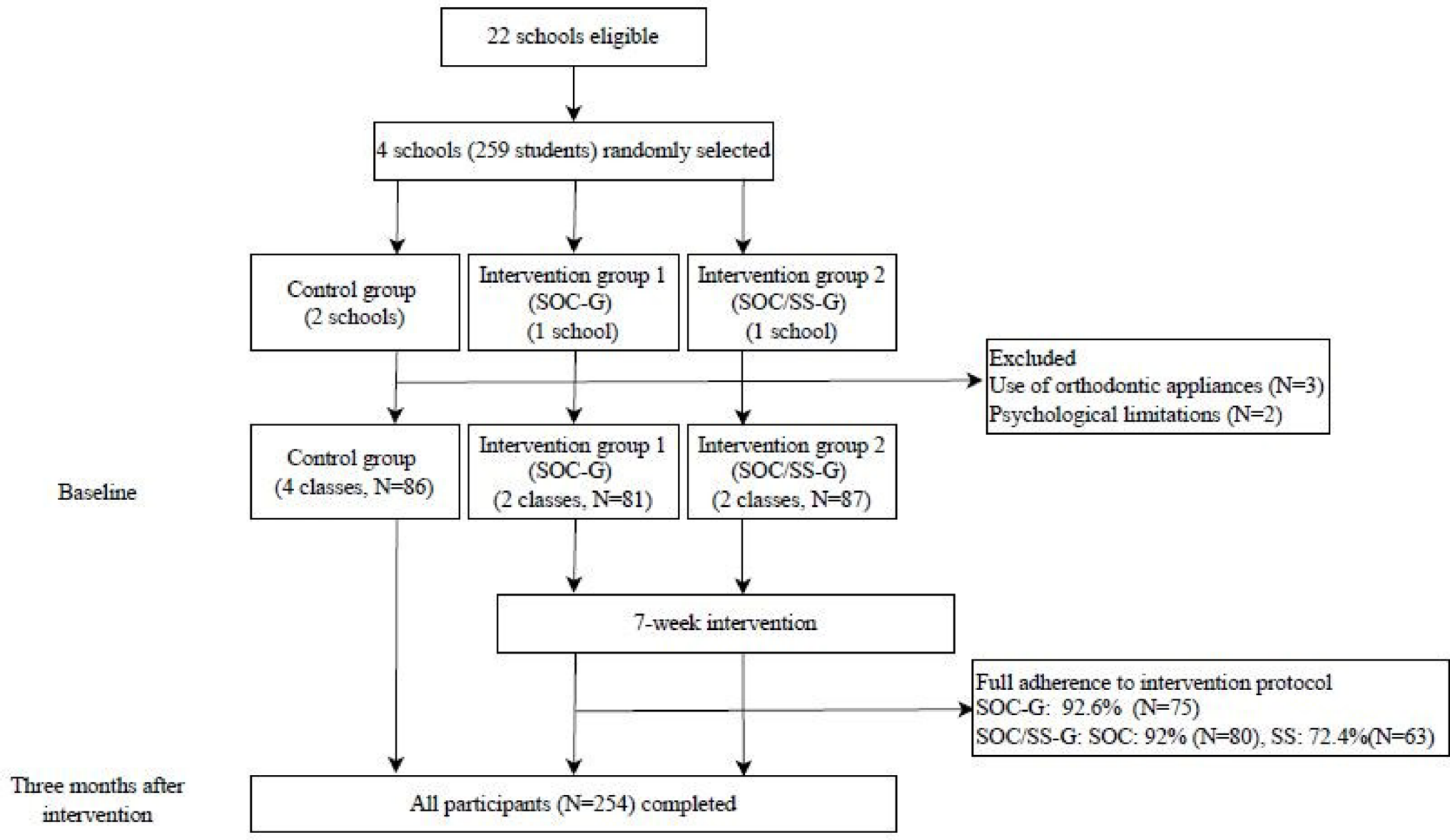
Flow diagram of participants

A total of 259 adolescents who were initially invited agreed to participate. Three students using orthodontic appliances and two students with psychological limitations that affected their understanding of the questionnaires were excluded. Therefore, 254 adolescents participated in the baseline and three months after intervention data collection (follow up rate = 100%): control group, *n* = 86; intervention group 1, *n* = 81; intervention group 2, *n* = 87 (Fig. 2). Half of the sample was composed of female adolescents, and the majority of participants’ mothers had between 1 and 7 years of schooling (53.5%). Demographic and socioeconomic characteristics, psychosocial factors, clinical data, and OHRQoL did not differ between participants in the three groups at baseline. At baseline, participants in the control group had better HRQoL than the intervention groups (Table 1).

### Effects of interventions

**Fig. 3:**
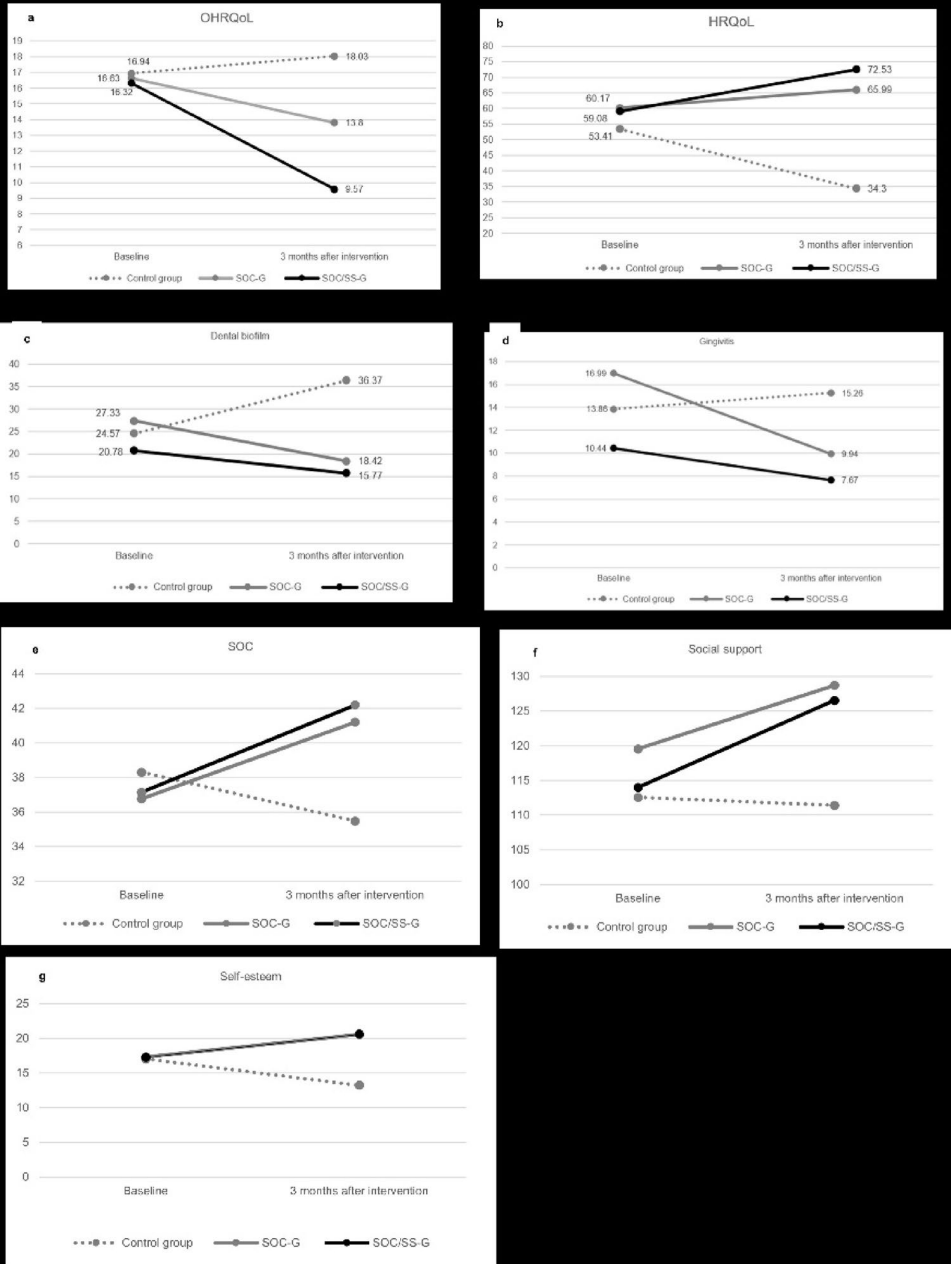
Mean scores of**a** OHRQoL, **b** HRQoL, **c** dental biofilm, **d** gingivitis, **e** SOC, **f** social support and **g** self-esteem between the three groups at baseline and 3 months after the interventions. OHRQoL, oral health-related quality of life, HRQoL, health-related quality of life SOC, sense of coherence, dental biofilm and gingivitis were assessed according to the number of sites with dental biofilm and number of sites with bleeding on probing measured at six sites per tooth, SOC-G: intervention group aimed to enhance adolescent’s sense of coherence, SOC/SS-G: intervention group aimed to enhance adolescent’s sense of coherence and social support.

SOC, social support, self-esteem, OHRQoL, HRQoL, dental biofilm, and gingivitis significantly improved between baseline and post-intervention evaluation in SOC-G and SOC/SS-G. SOC, self-esteem, HRQoL, dental biofilm and gingivitis significantly worsened in the control group (Table S2). Mixed-effects models showed significant intervention effects of SOC-G and SOC/SS-G on SOC, social support, self-esteem, dental biofilm, gingival status, OHRQoL, and HRQoL scores three months after interventions (Table S3 and Table S4). Psychosocial factors, clinical data and quality of life measures at baseline and three months after intervention are described in Fig. 3 and Table S5. Three months after intervention, SOC-G and SOC/SS-G had lower CPQ scores and higher Kiddo-KINDL scores than the control group, indicating better OHRQoL and HRQoL. No significant differences between OHRQoL and HRQoL were observed between SOC-G and SOC/SS-G three months after intervention. Adolescents from SOC-G and SOC/SS-G had CPQ scores 4.23 and 4.89 lower than those from control group. In addition, Kiddo-KINDL scores were 31.69 and 38.23 higher in SOC-G and SOC/SS-G groups than control group. SOC-G and SOC/SS-G also significantly increased SOC, social support, and self-esteem when compared to control group. Significant reductions on dental biofilm and gingivitis were detected in SOC-G and SOC/SS-G than control group after three months of intervention. Effect sizes for dental biofilm were 7.95 and 10.6 for SOC-G and SOC/SS-G, and 5.32 and 7.59 gingivitis for SOC-G and SOC/SS-G. Non-significant smaller effect sizes were observed between SOC-G and SOC/SS-G for dental biofilm (2.65) and gingivitis (2.27) (Table S5). The MID estimates for the SOC-G and SOC/SS-G were moderate to large or large for psychosocial factors, small or small to moderate for dental biofilm and gingivitis, and small to moderate or large for OHRQoL and HRQoL (Table S6).

**Table 3.** Standardised direct and indirect effects of the parsimonious structural equation model.

Direct effects	β	Bootstrap SE	Bias- Correct 95% CI	P
Dental biofilm (baseline) → gingivitis (after intervention)	0.729	0.054	0.615 / 0.826	0.002
Dental biofilm (baseline) → Dental biofilm (after intervention)	0.633	0.074	0.476 / 0.775	0.002
Intervention → Dental biofilm (after intervention)	−0.161	0.046	−0.246 / −0.068	0.002
Intervention groups → Self−esteem change	0.629	0.026	0.573 / 0.673	0.004
Intervention groups → SOC change	0.255	0.055	0.137 / 0.361	0.003
Intervention groups → Social support change	0.240	0.057	0.124 / 0.347	0.002
Intervention groups → HRQoL	0.650	0.042	0.559 / 0.728	0.003
Gingivitis (baseline) → Social support change	0.159	0.077	0.015 / 0.303	0.021
Dental biofilm (after intervention) → OHRQoL	0.150	0.065	0.030/ 0.294	0.010
Dental biofilm (after intervention) → gingivitis (after intervention)	0.737	0.067	0.582/ 0.853	0.003
Self-esteem change → HRQoL	0.259	0.040	0.188/ 0.335	0.002
SOC change → HRQoL	0.118	0.042	0.038/ 0.207	0.006
SOC change → OHRQoL	−0.331	0.073	−0.478/ −0.191	0.001
Social support change → gingivitis (after intervention)	−0.068	0.028	−0.134/ −0.020	0.005

Indirect effects	β	Bootstrap **SE**	Bias- Correct 95% CI	P
Dental biofilm (baseline) → Social support change	0.116	0.057	0.012/ 0.242	0.018
Dental biofilm (baseline) → OHRQoL	0.088	0.044	0.014/ 0.190	0.023
Dental biofilm (baseline) → gingivitis (after intervention)	0.459	0.070	0.322/ 0.599	0.002
Intervention groups → HRQoL	0.182	0.029	0.130/ 0.241	0.001
Intervention groups → OHRQoL	−0.149	0.049	−0.241/ −0.049	0.005
Intervention groups → gingivitis (after intervention)	−0.157	0.035	−0.235/ −0.092	0.001
Gingivitis (baseline) → Gingivitis (after intervention)	−0.011	0.006	−0.032/ −0.002	0.008

Adherence to full protocol in the SOC-G was reported by 92.6% of participants, while 7.4% attended six sessions. In the SOC/SSG-G, 92% adhered to the full SOC protocol, and 8% attended five sessions. Completion of the seven social support meetings was achieved by 72.4%, whereas 10.3% attended six sessions (Fig. 2). Reasons for non-attendance at social support meetings included lack of privacy when adolescents shared mobile phones with parents, low interest in responding to messages, and limited internet access.

### Structural equations

**Fig. 4:**
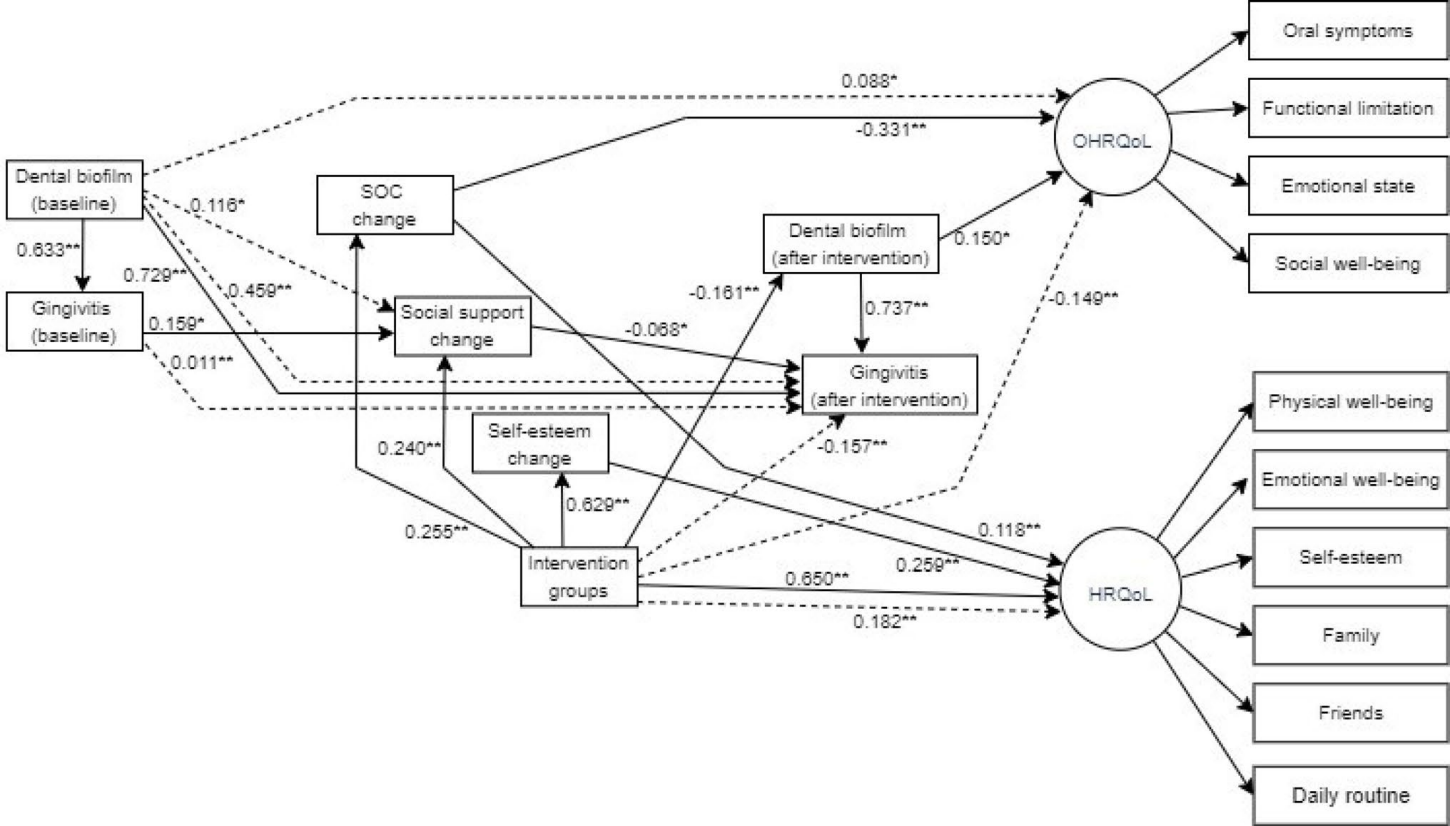
Significant direct and indirect effects for the parsimonious theoretical model. Nonsignificant paths have been deleted for ease of interpretation. SOC, sense of coherence. *P < 0.05. **P < 0.01

The measurement, full, and parsimonious models met all the model fit criteria (Table S6). Confirmatory factor analysis (CFA) supported the latent variables OHRQoL and HRQoL according to item loadings and 95% CIs (Fig. S1). The direct and indirect effects of interventions on primary and secondary outcomes are detailed using SEM (Fig. 4; Table 2). Intervention groups directly predicted improvements in SOC, social support and self-esteem after three months. Moreover, better HRQoL and lower dental biofilm were directly predicted by intervention groups. Social support change directly predicted lower gingivitis. Indirect effects of intervention groups on better OHRQoL, better HRQoL and lower gingivitis were also observed. SOC changes and self-esteem changes mediated the indirect effects of intervention groups on better HRQoL. Indirect effects of intervention groups on better OHRQoL were mediated by SOC changes and dental biofilm after intervention. Social support changes mediated the effect of intervention groups on gingivitis.

## Discussion

This study adopted the biopsychosocial model to test the impact of two different interventions aimed at enhancing SOC and SOC along with social support on quality of life, dental biofilm, and gingival status in students aged 15 to 16 years. The present findings support the hypothesis that strengthening psychosocial factors during adolescence can positively influence oral health and quality of life outcomes. Adolescents who received interventions to improve SOC alone and those exposed to intervention to improve both SOC and social support showed substantial improvements on SOC, social support, and self-esteem three months after the interventions. These interventions also resulted in better OHRQoL and HRQoL for adolescents, and significant decline in dental biofilm and gingivitis compared to those who did not receive any intervention. However, the intervention targeting both SOC and social support did not result in additional improvements in quality of life, gingival status, and dental biofilm when compared with the intervention aiming to enhance SOC. According to SEM analysis, the interventions had a direct and positive effect on participant’s SOC, social support and self-esteem. In addition, HRQoL and dental biofilm were directly influenced by the interventions. The positive impact of interventions on OHRQoL was indirect, mediated via SOC change and lower dental biofilm, while interventions indirectly predicted a decline in gingivitis through changes in social support (Table 3).

Our study compared a previously tested SOC intervention [6, 7] with an innovative intervention designed to enhance adolescents’ SOC and social support. The present study demonstrated that strengthening individuals’ SOC, or both SOC and social support results in better oral health. Our findings support the general resistance resources theory and salutogenic model as a conceptual framework to develop oral health promotion interventions to improve children and adolescent’s oral health [23, 24]. The interventions investigated may have influenced the clinical measures and quality of life through the following mechanisms. The psychological pathway whereby enhancing SOC and social support would facilitate the development of coping strategies to deal with life stressors and to promote health [23, 41]. Moreover, according to our findings, self-esteem significantly increased in the intervention groups and was a relevant pathway of the impact of psychosocial interventions on HRQoL. This finding is supported by previous evidence on the association of self-esteem with self-efficacy, life satisfaction, and HRQoL in adolescents [42, 43]. Intervention to improve SOC can also attenuate oral conditions symptoms as a result of a positive shift in the appraisal of external stimulus where the individual feels life more structured, manageable, meaningful, and worthy of investment and engagement [23]. Current evidence on the influence of SOC and social support on frequency of toothbrushing, dietary habits and dental attendance supports the behavioural pathway between SOC and support changes and oral health [44, 45]. In this study, the mediation effect of dental biofilm on the influence of intervention groups on gingivitis and OHRQoL endorses the behavioural pathway. This finding is supported by previous research showing that self-esteem was indirectly associated with BOP through toothbrushing frequency and oral hygiene effectiveness [13].

Previous systematic reviews highlighted the limitations of preventive interventions approaches to improve children’s and adolescent’s oral health, including the focus on oral health education and the lack of a theoretical basis for intervention development [3, 16]. They also concluded that theory-based psychological and social interventions, such as self-efficacy theory and social-cognitive theory, can result in greater effectiveness in oral health promotion interventions [3, 16]. The main novelty of this experimental study was the evaluation of a new intervention combining strategies to enhance adolescents’ SOC and social support. The new intervention resulted in improvements in all psychosocial factors, quality of life outcomes, and clinical measures. However, the influence on psychosocial factors and oral health outcomes did not significantly differ from the intervention focused on enhancing SOC. This was an unexpected finding, as it was hypothesised that adding the social support intervention component would provide an additional benefit to oral health outcomes. A possible explanation is that both interventions adopted active, person-centred approaches through participatory, engaging, and empowering methods to improve and maintain oral health. Therefore, the intervention aimed at improving SOC alone was also capable of enhancing adolescents’ social support. Moreover, it can be argued that the quality of social support provided in the SOC/SS-G was insufficient to add significant benefits to the investigated outcomes. In addition, the intensity and duration of the social support intervention appear to be lower than necessary to result in additional benefits for primary and secondary outcomes. Thus, future intervention studies on social support should assess the quality of interactions using qualitative methods. The three-month follow-up period may not provide enough time to detect the additional benefits of the social support intervention. Sustained social support interventions over a longer period might be necessary to observe significant benefits on oral health.

Our findings indicate that oral health promotion strategies grounded in the salutogenic model and peer social support have the potential to replace traditional education-focused approaches. Future policies should prioritise scalable, theory-driven programmes integrated into schools and communities. The short-term benefits reported in this study highlight the need for longer-term, sustained interventions incorporating reinforcement strategies such as booster sessions and strengthening peer-support networks. Robust monitoring and evaluation systems are essential for scaling and ensuring effectiveness, and these should be adapted to different socioeconomic contexts to promote equity.

This study has some limitations to be addressed. This study included students aged 15 to 16 years recruited from socially vulnerable communities. Thus, generalisation of the current findings to other age groups and adolescents from better socioeconomic backgrounds are not applicable. The long-term benefits of interventions were not evaluated, as the outcomes were assessed only three months after the interventions. School dropouts are relatively high among socially deprived adolescents. Therefore, a high loss to follow up was anticipated if post-intervention assessment interval was longer than three months. Gingivitis and dental biofilm measures may fluctuate over a three-month period and might not be considered the ideal outcomes. Future studies should consider behavioural indicators as secondary measures. The quality of peer interactions within the SOC/SS-G was not assessed, representing both a limitation and an opportunity for improvement in future studies aimed at elucidating the mechanisms of social support. Some participants did not fully adhere to the intervention protocols, which may have partially influence the findings. Future studies should be conducted in children and adolescents from middle and high socioeconomic groups and evaluate the impact of interventions for longer periods. Additionally, future clinical trials are encouraged to address other relevant oral health outcomes.

## Conclusion

This study provides evidence that enhancing psychosocial factors during adolescence can improve oral health and quality of life outcomes. Adolescents who participated in interventions aimed at improving SOC, as well as those who received combined interventions to enhance both SOC and social support, showed substantial improvements in SOC, social support, and self-esteem three months post-intervention. These psychosocial enhancements were associated with significant improvements in OHRQoL and HRQoL, along with meaningful reductions in dental biofilm and gingivitis.

## Supplementary Information

Below is the link to the electronic supplementary material.

Supplementary Material 1
