# Supplementary material for: Interventions to improve adolescents’ sense of coherence and social support on quality of life and gingivitis: a cluster-randomised clinical trial
Source: Qual Life Res. 2026 Jun 5;35(7):161. doi: 10.1007/s11136-026-04232-w (PMC13241411; doi:10.1007/s11136-026-04232-w)

Table S1. Internal consistency of questionnaires.

| Construct | Instrument | Baseline | Three months after intervention |
| --- | --- | --- | --- |
| Sense of coherence | SOC-13 | 0.906 | 0.813 |
| Social Support | SSA | 0.897 | 0.889 |
| Self-esteem | RSES | 0.845 | 0.847 |
| OHRQoL | CPQ 11-14 | - | 0.833 |
| HRQoL | Kiddo-KINDL | - | 0.808 |

Table S2. Pairwise comparisons of SOC, social support and self-esteem within groups between baseline and 3-month post intervention.

| Groups and variables | Baseline | 3 months after intervention | *P* |
| --- | --- | --- | --- |
|  | Mean (SD) | Mean (SD) |  |
| Control group |  |  |  |
| SOC | 38.3 (7.54) | 35.48 (8.21) | 0.008 |
| Social Support | 112.56 (19.21) | 111.40 (22.25) | 0.450 |
| Self-esteem | 17.03 (5.02) | 13.23 (3.23) | < 0.001 |
| OHRQoL | 16.94 (9.83) | 18.03 (8.41) | 0.123 |
| HRQoL | 53.41 (11.88) | 34.30 (9.49) | < 0.001 |
| Dental biofilm | 24.57 (26.78) | 26.37 (28.13) | 0.001 |
| Gingivitis | 13.86 (21.71) | 15.26 (23.30) | < 0.001 |
| SOC-G |  |  |  |
| SOC | 36.77 (8.19) | 41.20 (8.86) | 0.005 |
| Social Support | 119.54 (16.92) | 128.70 (20.23) | 0.002 |
| Self-esteem | 17.28 (6.00) | 20.54 (4.89) | < 0.001 |
| OHRQoL | 16.63 (9.45) | 13.80 (8.60) | 0.003 |
| HRQoL | 60.17 (13.28) | 65.99 (12.96) | < 0.001 |
| Dental biofilm | 27.33 (22.94) | 18.42 (16.41) | < 0.001 |
| Gingivitis | 16.99 (24.25) | 9.94 (16.81) | < 0.001 |
| SOC/SS-G |  |  |  |
| SOC | 37.15 (8.21) | 42.20 (7.05) | < 0.001 |
| Social Support | 113.98 (13.37) | 128.70 (20.23) | < 0.001 |
| Self-esteem | 17.25 (5.22) | 20.56 (4.46) | < 0.001 |
| OHRQoL | 16.48 (10.78) | 13.14 (7.77) | 0.003 |
| HRQoL | 59.08 (13.37) | 72.53 (8.10) | < 0.001 |
| Dental biofilm | 20.78 (18.85) | 15.77 (16.56) | < 0.001 |
| Gingivitis | 10.44 (16.42) | 7.67 (13.76) | 0.002 |

Table S3. Type III Tests of fixed effects for sense of coherence, social support, self-esteem, OHRQoL, HRQoL, dental biofilm, and gingivitis three months after intervention.

| Source | df | F | *P* |
| --- | --- | --- | --- |
| ***Sense of coherence*** |  |  |  |
| Intercept | 1 | 317.176 | < 0.001 |
| Intervention groups | 2 | 16.833 | < 0.001 |
| Sense of coherence at baseline | 1 | 2.264 | 0.134 |
| ***Social support*** |  |  |  |
| Intercept | 1 | 166.336 | < 0.001 |
| Group | 2 | 17.441 | < 0.001 |
| Social support at baseline | 1 | 8.711 | 0.003 |
| ***Self-esteem*** |  |  |  |
| Intercept | 1 | 189.269 | < 0.001 |
| Intervention groups | 2 | 226.118 | < 0.001 |
| Self-esteem at baseline | 1 | 450.534 | 0.003 |
| ***OHRQoL*** |  |  |  |
| Intercept | 1 | 108.933 | < 0.001 |
| Intervention groups | 2 | 9.780 | < 0.001 |
| OHRQoL at baseline | 1 | 42.894 | < 0.001 |
| ***HRQoL*** |  |  |  |
| Intercept | 1 | 193.358 | < 0.001 |
| Intervention groups | 2 | 349.700 | < 0.001 |
| HRQoL at baseline | 1 | 58.383 | < 0.001 |
| ***Dental biofilm*** |  |  |  |
| Intercept | 1 | 15.282 | < 0.001 |
| Intervention groups | 2 | 8.894 | < 0.001 |
| Dental biofilm at baseline | 1 | 182.685 | < 0.001 |
| ***Gingivitis*** |  |  |  |
| Intercept | 1 | 5.583 | 0.019 |
| Intervention groups | 2 | 7.337 | 0.001 |
| Gingivitis at baseline | 1 | 265.556 | < 0.001 |

Table S4. Estimates of fixed effects for sense of coherence, social support, self-esteem, OHRQoL, HRQoL, dental biofilm, and gingivitis 3 months after intervention.

|  | Estimate | Std error | *t* | *P* | 95% confidence interval |
| --- | --- | --- | --- | --- | --- |
| ***Sense of coherence*** |  |  |  |  |  |
| Intercept | 39.114 | 2.569 | 15.228 | < 0.001 | 34.055 / 44.173 |
| Control | 0 | 0 |  |  |  |
| SOC-G | 5.599 | 1.248 | 4.487 | < 0.001 | 3.141 / 8.057 |
| SOC/SS-G | 6.634 | 1.224 | 5.418 | < 0.001 | 4.223 / 9.045 |
| Sense of coherence at baseline | -0.096 | 0.064 | -1.505 | 0.134 | -0.221 / 0.030 |
| ***Social support*** |  |  |  |  |  |
| Intercept | 89.454 | 7.715 | 11.595 | < 0.001 | 74.260 / 104.648 |
| Control | 0 | 0 |  |  |  |
| SOC-G | 15.298 | 2.998 | 5.103 | < 0.001 | 9.393 / 21.203 |
| SOC/SS-G | 14.845 | 2.910 | 5.102 | < 0.001 | 9.115 / 20.576 |
| Social support at baseline | 0.195 | 0.066 | 2.951 | < 0.001 | 0.065 / 0.325 |
| ***Self-esteem*** |  |  |  |  |  |
| Intercept | 2.554 | 0.573 | 4.454 | < 0.001 | 1.424 / 3.683 |
| Control | 0 | 0 |  |  |  |
| SOC-G | 7.143 | 0.393 | 18.159 | < 0.001 | 6.368 / 7.917 |
| SOC/SS-G | 7.182 | 0.386 | 18.593 | < 0.001 | 6.421 / 7.943 |
| Self-esteem at baseline | 0.628 | 0.030 | 21.226 | < 0.001 | 0.569 / 0.686 |
| ***OHRQoL*** |  |  |  |  |  |
| Intercept | 12.709 | 1.158 | 10.972 | < 0.001 | 10.427 / 14.990 |
| Control | 0 | 0 |  |  |  |
| SOC-G | -4.134 | 1.184 | -3.491 | 0.001 | -6.467 / -1.802 |
| SOC/SS-G | -4.753 | 1.163 | -4.086 | < 0.001 | -7.044 / -2.462 |
| OHRQoL at baseline | 0.314 | 0.048 | 6.549 | < 0.001 | 0.220 / 0.409 |
| ***HRQoL*** |  |  |  |  |  |
| Intercept | 15.636 | 2.641 | 5.919 | < 0.001 | 10.434 / 20.839 |
| Control | 0 | 0 |  |  |  |
| SOC-G | 29.335 | 1.475 | 19.882 | < 0.001 | 26.439 / 32.240 |
| SOC/SS-G | 36.249 | 1.440 | 25.166 | < 0.001 | 33.412 / 39.086 |
| HRQoL at baseline | 0.349 | 0.0.46 | 7.641 | < 0.001 | 0.259 / 0.440 |

Fig. S1 Measurement model


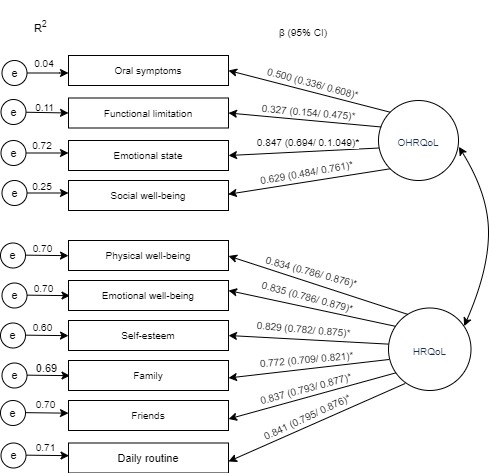

Supplement: Supplementary file 1 — Supplementary Material 1 [file 11136_2026_4232_MOESM1_ESM.docx]
